# Artesunate promotes Th1 differentiation from CD4^+^ T cells to enhance cell apoptosis in ovarian cancer via miR-142

**DOI:** 10.1590/1414-431X20197992

**Published:** 2019-04-29

**Authors:** Xiao Chen, Xue-ling Zhang, Guo-hua Zhang, Ying-fang Gao

**Affiliations:** Department of Gynecology and Obstetrics, The Fourth Hospital of Shijiazhuang, Shijiazhuang, China

**Keywords:** Artesunate, Ovarian cancer, CD4^+^ T cells, Th1 cells, miR-142, Sirt1

## Abstract

The aim of this study was to evaluate the influence of artesunate on Th1 differentiation and its anti-tumor effect on ovarian cancer. A Murine ovarian cancer model was established by ID8 cells transplantation. The expression of miR-142 and Sirt1 proteins in peripheral CD4^+^ T cells were quantified with qRT-PCR and western blot, respectively. Peripheral CD4^+^ T cells were induced for Th1 differentiation. The percentages of apoptosis of Th1/CD4^+^ T cells and ovarian cancer cells were analyzed by flow cytometry. The IFN-γ level was examined through enzyme-linked immunosorbent assay. Artesunate promoted miR-142 expression in peripheral CD4^+^ T cells and Th1 differentiation from CD4^+^ T cells. Artesunate promoted cell apoptosis of ovarian cancer cells by inducing Th1 differentiation. By up-regulating miR-142, artesunate suppressed Sirt1 level and promoted Th1 differentiation. Artesunate enhanced the pro-apoptotic effects of Th1 cells on ovarian cancer via the miR-142/Sirt1 pathway. Artesunate promoted Th1 differentiation from CD4^+^ T cells by down-regulating Sirt1 through miR-142, thereby enhancing cell apoptosis in ovarian cancer.

## Introduction

Although relatively uncommon, ovarian cancer is the most lethal gynecological cancer affecting women all over the world (1,2). Most patients are diagnosed with advanced disease and usually treated with surgical resection followed by platinum-based chemotherapy, but the relapse rate is rather high and the survival rate improved just slightly in recent decades (3,4). Novel effective therapies are urgently needed for improving ovarian cancer outcomes. Promisingly, artesunate sensitized ovarian cancer cells to cisplatin, exhibiting its potential anti-tumor role in ovarian cancer (5). The action mechanism of artesunate in defending against ovarian cancer deserves further clarification.

Artesunate is one of the semi-synthetic derivatives of artemisinin that has good water solubility and antimalarial effects (6). In addition, artemisinin and its derivatives possess anti-tumor activities, anti-inflammatory functions, and anti-fibrotic effects in diverse diseases (7–9). For instance, artesunate induced cell apoptosis in lung adenocarcinoma and esophageal cancer (10,11), and it induced radiosensitivity in cervical cancer cells (12). In addition, immunomodulatory effects of artesunate in cancer have been reported (13), implying its effect as a drug for immunotherapy. It has been demonstrated that artesunate restrained the proliferation of CD4^+^ T cells but enhanced the function of interferon (IFN)-γ-producing CD4^+^ T (Th1) cells to secret IFN-γ (14). It can be inferred that artesunate has a significant influence on Th1 differentiation from CD4^+^ T cells, while the underlying mechanism remains uncertain.

By producing various cytokines including IFN-γ, tumor necrosis factor (TNF)-β, and interleukin (IL)-2, Th1 cells show a strong effect in repressing tumor growth, and IFN-γ possesses capabilities for anti-tumor effects and immunomodulation (15). However, enhanced expression and activity of the Silence information regulator 1 (Sirt1) caused by resveratrol suppressed CD4^+^ T cells activation and IFN-γ production (16), suggesting that expression and activity of Sirt1 played a negative role in Th1 differentiation and IFN-γ secretion. In serous epithelial ovarian cancer, over-expression of Sirt1 contributes to chemoresistance and poor prognosis (17). Additionally, Sirt1 has been identified as a target of microRNA-142 (miR-142) (18), increasing its expressions in the ascites and sera of ovarian cancer patients (19). Moreover, miR-142 also attenuated the migration of CD4^+^ T cells (20). Given that artesunate positively or negatively regulated miRNAs expression (21,22), we proposed the hypothesis that artesunate may down-regulate Sirt1 through miR-142 to promote Th1 differentiation from CD4^+^ T cells, thus enhancing its anticancer effect against ovarian cancer. Therefore, this study was carried out to elucidate the impact and regulatory mechanism of artesunate on ovarian cancer cell apoptosis, aiming to seek strategies for improving treatment outcomes of ovarian cancer.

## Material and Methods

### Ovarian cancer cells culture

The mouse ovarian cancer cell line, ID8, was grown in complete Roswell Park Memorial Institute medium (RPMI-1640) supplemented with 10% fetal bovine serum (FBS), 4 mM L-glutamine, 20 mM HEPES, 0.1 mM 2-mercaptoethonal, 100 IU/mL penicillin, and 100 μg/mL streptomycin at 37°C and 5% CO_2_. ID8 cells were seeded into 96-well plates until cell growth was observed. Outgrown cells were then trypsinized and washed for cell suspension preparation before being transplanted to mice.

### Establishment of ovarian cancer model

This study was approved by the Animal Care Committee of the Fourth Hospital of Shijiazhuang. All protocols of animal experiments were performed according to the Guide for the Care and Use of Laboratory Animals proposed by the Chinese National Institutes of Health. C57BL/6 mice were injected intra-peritoneally (*ip*) with 2×10^6^ ID8 cells suspended in 200 μL sterile PBS. Thirty days after ID8 injection, mice were randomly injected with artesunate at 4 concentrations: 0, 10, 50, and 100 mg/kg (n=8 in each group). Twelve days after injection, peripheral blood of the mice was collected for CD4^+^ T cells isolation. After sacrifice, the tumor tissue was excised for volume measurement.

### Isolation of peripheral CD4^+^ T cells

CD4^+^ T cells were isolated from the peripheral blood with the magnetic bead cell sorting (MACS) method. Briefly, the peripheral blood mononuclear cells (PBMCs) were isolated using lymphocyte separation medium, and washed with PBS. After centrifugation at 800 *g* for 15 min at 4°C, the supernatant was removed, and the PBMCs were re-suspended with MACS buffer. After the antibodies were tagged with biotin, the mixtures were incubated in the dark for 10 min at 4°C. Then, the antibiotic beads, MACS buffer, and PE-CD25 McAb were added and incubated in the dark for 15 min at 4°C. Finally, the cells were washed and re-suspended with 500 μL MACS buffer to obtain the cell suspension, which was added into the LD column (Miltenyi Biotec, Germany). Cells that flowed through the column, CD4^+^ T cells, were collected and evaluated with flow cytometry. More than 96% of purified cells were identified as CD4-expressing T cells.

### Isolation of tumor-infiltrating lymphocytes

In order to explore the effect of artesunate on lymphocyte activity in the tumor microenvironment, we isolated the tumor-infiltrating lymphocytes from solid tumor samples of ovarian cancer in mice. The tumor tissue was mechanically minced into 1 mm^3^, washed with RPMI-1640 medium, and then incubated in RPMI-1640 with 0.14% collagenase type I and 0.01% DNAse in a magnetic stirring apparatus (RO 10, IKA, Germany) overnight at 4°C. After filtration through a 150-μm Nylon mesh, the single cell suspension was washed in RPMI-1640 medium containing 10% autologous plasma and placed on discontinuous Ficoll-Hypaque (Sigma, USA) density gradients. Finally, the tumor-infiltrating lymphocytes were harvested after centrifugation at 400 *g* for 20 min at room temperature. The Th1/CD4^+^ T percentage was analyzed with flow cytometry using a flow cytometer (Becton Dickinson, USA).

### Quantitative real-time PCR (qRT-PCR)

Total RNA was extracted from CD4^+^ T cells using the RNeasy Plus Mini Kit (Qiagen, USA) according to the supplier's manual. The first‐strand cDNA was synthesized using M-MLV Reverse Transcriptase Kit (Thermo Fisher, USA) based on the manufacturer’s protocol. QRT-PCR was performed with the SYBR Select Master Mix (Thermo Fisher) and analyzed on an ABI 7900-fast thermocycler (Applied Biosystems, USA). Relative expression of miR-142 was normalized with U6, and the relative expression of Sirt1 mRNA was normalized to GAPDH. The comparative Ct (ΔΔCt) method was used for quantification. The primers used for qPCR were designed and synthesized by Sangon Biotech (China). The primer sequence of miR-142 was F: 5′-AACTCCAGCTGGTCCTTAG-3′; R: 5′-TCTTGAACCCTCATCCTGT-3′ and of Sirt1 was F: 5′-CTGTTTCCTGTGGGATACCTGACT-3′; R: 5′-ATCGAACATGGCTTGAGGATCT-3′.

### Flow cytometry

For Th1/CD4^+^ T cells percentage analysis, CD4^+^ T cells were collected and activated with PMA (50 ng/mL) for 2 h, and then monensin (3 μM, a transport inhibitor) was added for an additional 2-h incubation. After harvesting and washing with PBS, CD4^+^ T cells were permeabilized with permeabilization solution (BD Biosciences, USA) for 10 min and fixed with 4% paraformaldehyde for 20 min. For staining, PE-conjugated anti-human IFN-γ antibody (BD Biosciences) was added to cells for 30 min and washed with PBS containing 0.5% FBS. The stained cells were subjected to flow cytometric analysis on a FACSCalibur cytometer (BD Biosciences) and analyzed via CELLQuest software (BD Biosciences).

For apoptosis analysis, ID8 cells were collected and incubated in an annexin V-FITC/propidium iodide (PI) cell apoptosis detection kit (Sigma, USA). Briefly, cells were resuspended with 200 μL binding buffer and incubated with 5 μL annexin V (conjugated with FITC or APC) in the dark for 15 min at 37°C. Finally, the cells were stained with PI or V450 at RT for 15 min, followed by flow cytometric analysis using a FACSCalibur flow cytometer and CellQuest software (BD Biosciences).

### Western blot

Western blot determination was performed to display the protein level in CD4^+^ T cells. Briefly, total proteins were extracted from CD4^+^ T cells using RIPA lysis buffer (containing a protease inhibitor cocktail). Then, the protein extracts were subjected to 10% SDS-PAGE and transferred to PVDF membrane. After being blocked with 5% non-fat milk for 1 h at room temperature, the membrane was incubated using the primary antibodies including anti-Sirt1 and anti‐β-actin (Abcam, UK) at 4°C overnight, followed by secondary antibody at room temperature for 2 h. Bands were visualized by ECL (GE Healthcare, Sweden).

### CD4^+^ T cells culture and Th1 differentiation induction

CD4^+^ T cells (1×10^7^ cells/mL) were cultured in complete RPMI1640 medium in the presence of 10% fetal bovine serum (FBS), 1% penicillin-streptomycin, and 10 mM HEPES. After activation with plate-bound 1 μg/mL anti-CD3 antibody and μg/mL anti-CD28 antibody, CD4^+^ T cells were stimulated with 50 U/mL rhIL-2, 10 μg/mL anti-IL-4 antibody, and 3 ng/mL rIL-12 (Bioscience, USA) for Th1 cell differentiation.

### Enzyme-linked immunosorbent assay (ELISA)

ELISA system kits (eBioscience, USA) were used to evaluate the level of IFN-γ in CD4^+^ T cell supernatants. ELISA plates were coated with monoclonal antibody specific to IFN-γ (100 μL/well), washed, and incubated with blocking buffer overnight at 4°C. The supernatant of CD4^+^ T cells culture was added and incubated at 37°C for 2 h at room temperature. After washing, biotinylated anti-IFN-γ (Biosynthesis Biotechnology, China) was added for incubation. The plates were washed and incubated with streptavidin-peroxidase. One hundred microliters of tetramethylbenzidine (Sigma) substrate was added and developed for 10 min, which was terminated with 2 M sulfuric acid (Sigma). The absorbance was measured at 450 nm using a microplate ELISA reader (Molecular Devices, USA).

### Cell transfection

To evaluate the effect of miR-142 or Sirt1 expression on Th1 differentiation, vectors including miR-142 inhibitor, si-Sirt1, or their negative control were purchased from GenePharma Co. Ltd (China). CD4^+^ T cells were transfected or co-transfected with these vectors using Lipofectamine2000 (Invitrogen, USA) according to the manufacturer's instructions.

### Statistical analysis

SPSS 21.0 software (IBM, USA) was applied for statistical analyses. All experiments were repeated at least three times, and the data are reported as means±SD. Differences between groups were analyzed with Student's *t*-test (between two groups) or one-way ANOVA (for more than two groups), and a value of P<0.05 was considered significant.

## Results

### Artesunate promoted Th1 differentiation and miR-142 expression

**Figure 1.**
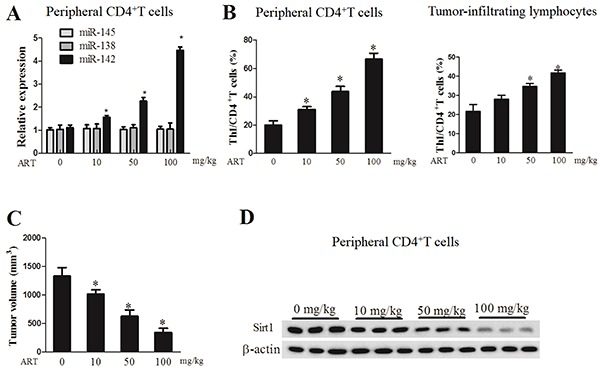
Artesunate (ART) promoted Th1 differentiation and miR-142 expression. **A**, Expression of miRNAs was quantified by qRT-PCR. **B**, Percentage of Th1/CD4^+^T cells in the peripheral CD4^+^T cells and in the tumor-infiltrating lymphocytes was analyzed with flow cytometry. **C**, Tumor tissue of mice was excised for volume measurement. **D**, Sirt1 protein level was determined with western blot. Data are reported as means±SD. *P<0.05 compared to 0% ART (ANOVA).

The expression detection of miRNAs in peripheral CD4^+^ T cells indicated that only the miR-142 level was dose-dependently elevated with the increasing artesunate concentration, while no significant difference was noted in the miR-145 and miR-138 expression levels (Figure 1A). As the artesunate concentration increased, the percentage of the Th1/CD4^+^ T cells in the peripheral CD4^+^ T cells rose in a dose-dependent manner, and the Th1/CD4^+^ T proportion in the tumor-infiltrating lymphocytes was elevated along with the artesunate concentration (Figure 1B). The tumor volume decreased as the artesunate concentration increased, and was the smallest in mice treated with 100 mg/kg artesunate (Figure 1C). The expression level of Sirt1 protein in peripheral CD4^+^ T cells was reduced in a dose-dependent manner (Figure 1D). These results revealed that artesunate promoted Th1 differentiation of peripheral CD4^+^ T cells, activated lymphocytes in the tumor microenvironment, and upregulated miR-142 expression in peripheral CD4^+^ T cells of ovarian cancer mouse model.

### Artesunate promoted apoptosis of ovarian cancer cells by inducing Th1 differentiation

**Figure 2.**
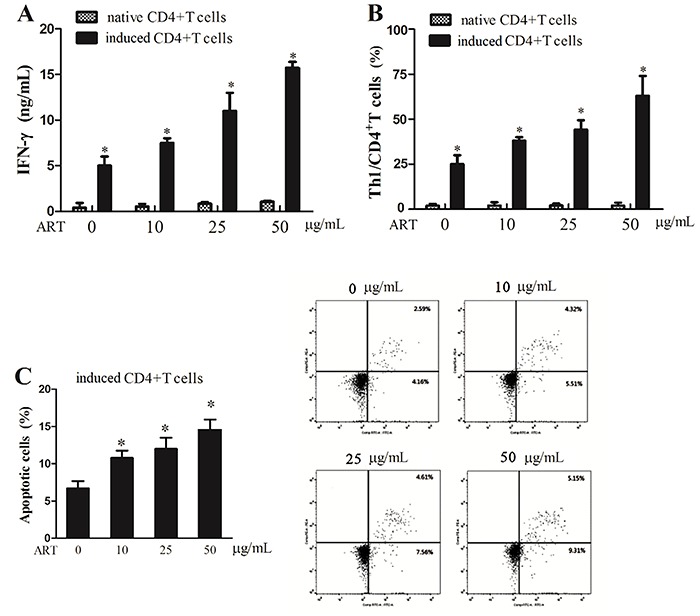
Artesunate (ART) promoted cell apoptosis of ovarian cancer through inducing Th1 differentiation. **A**, Interferon (IFN)-γ level in culture supernatant was examined by enzyme linked immunosorbent assay. **B,** Percentage of Th1/CD4^+^T cells was analyzed using flow cytometry. **C**, After co-cultured with the differentiation-induced and artesunate-treated CD4+T cells, ID8 cells apoptosis was measured using flow cytometry. Data are reported as means±SD. *P<0.05 compared to native CD4^+^ T cells (A and B) and to 0% ART (ANOVA).

As the influence of artesunate on Th1 differentiation and miR-142 expression in ovarian cancer mouse model is known, its influence on ovarian cancer cells was evaluated. Compared with 0 μg/mL, the IFN-γ level was markedly higher in cells treated with 50 μg/mL artesunate, and the level of IFN-γ increased as the concentration increased, while no significant change in IFN-γ levels was observed in the naïve CD4^+^ T cells (Figure 2A). The Th1/CD4^+^ T cells percentage was also elevated with the increased artesunate concentration, but the Th1/CD4^+^ T proportion in the naïve CD4^+^ T cells remained unchanged (Figure 2B). The differentiation-induced and artesunate-treated CD4^+^ T cells were then co-cultured with mouse ovarian cancer cell line ID8. After being cultured for 5 days, ID8 cells apoptosis was assessed. As shown in Figure 2C, artesunate accelerated apoptosis in ovarian cancer cells in a dose-dependent way. We illustrated that artesunate promoted cell apoptosis of ovarian cancer by inducing Th1 differentiation.

### Artesunate suppressed Sirt1 level by up-regulating miR-142 to induce Th1 differentiation

**Figure 3.**
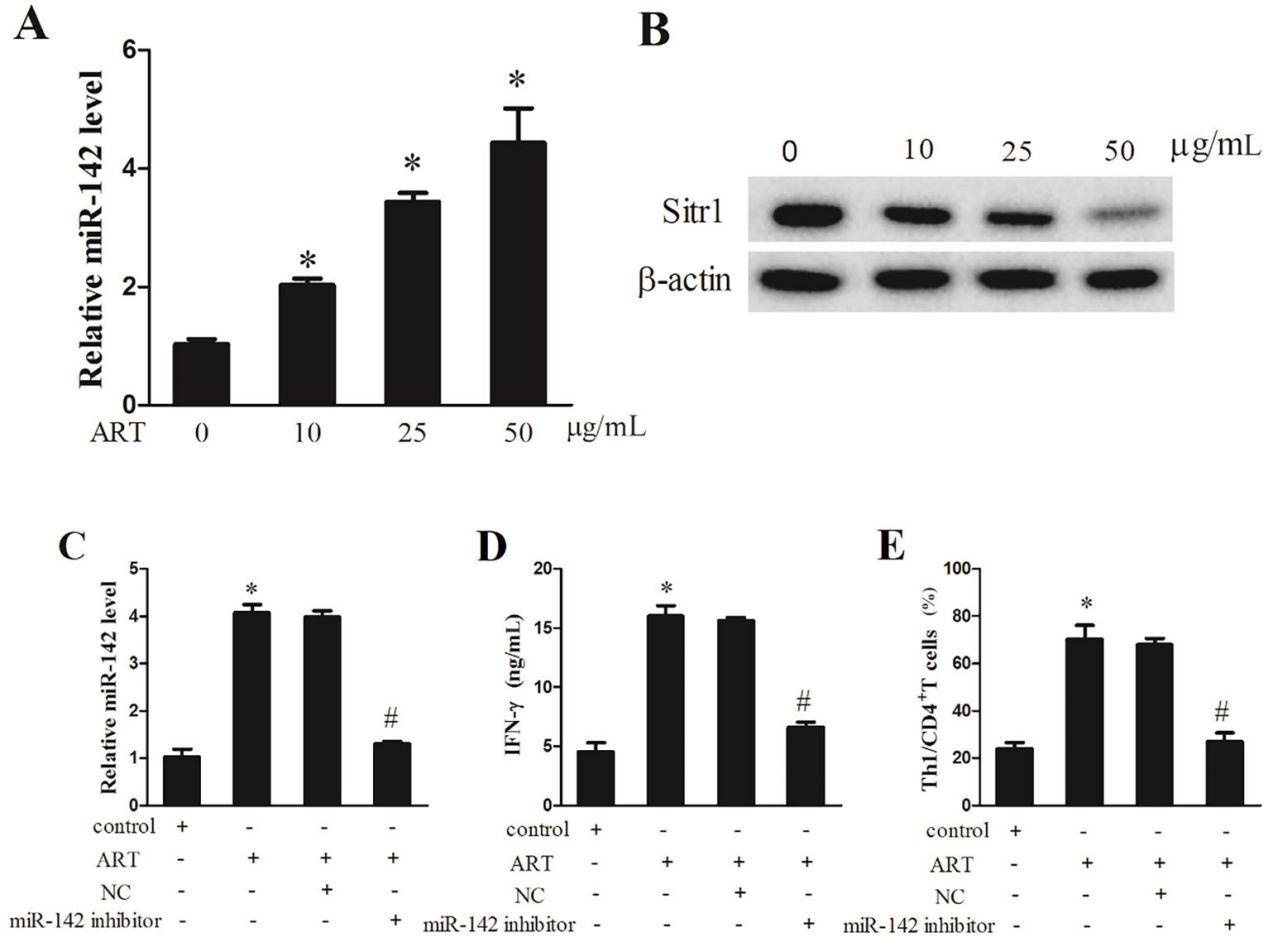
Artesunate (ART) suppressed Sirt1 level by up-regulating miR-142 to induce Th1 differentiation. **A**, Expression of miR-142 was detected by qRT-PCR. **B**, Sirt1 protein level was determined using western blot. CD4^+^ T cells were transfected with miR-142 inhibitor or its negative control (NC). **C**, Expression of miR-142 was determined by qRT-PCR. **D**, The IFN-γ level in culture supernatant was examined by ELISA. **E**, The proportion of Th1/CD4^+^ T cells was analyzed using flow cytometry. Data are reported as means±SD. *P<0.05 compared to 0% ART (**A**) or to control (**C**-**E**); ^#^P<0.05 compared to ART+NC (ANOVA).

To explore the mechanism underlying the artesunate regulation of Th1 differentiation, CD4^+^ T cells were induced for Th1 differentiation and then treated with artesunate. The expression of miR-142 was increased by artesunate, and the highest miR-142 level was noted in CD4^+^ T cells treated with 50 μg/mL artesunate (Figure 3A), while the Sirt1 protein level was lowered by artesunate in a dose-dependent manner (Figure 3B). After induction for Th1 differentiation from CD4^+^ T cells and treatment with artesunate (ART, 50 μg/mL), CD4^+^ T cells were transfected with miR-142 inhibitor or its negative control (NC) and assigned into 4 groups: control, ART, NC, and miR-142 inhibitor. Compared with the control group, artesunate enhanced miR-142 expression, but miR-142 inhibitor reversed its high expression (Figure 3C). The IFN-γ level in supernatant was increased with artesunate treatment but lowered by miR-142 inhibitor (Figure 3D), and the Th1/CD4^+^ T cells percentage was also increased after artesunate treatment but reduced with miR-142 inhibitor transfected (Figure 3E). Herein, we showed that artesunate suppressed Sirt1 level and induced Th1 differentiation by up-regulating miR-142.

### Artesunate enhanced the pro-apoptotic effects of Th1 cells on ovarian cancer via the miR-142/Sirt1 pathway

**Figure 4.**
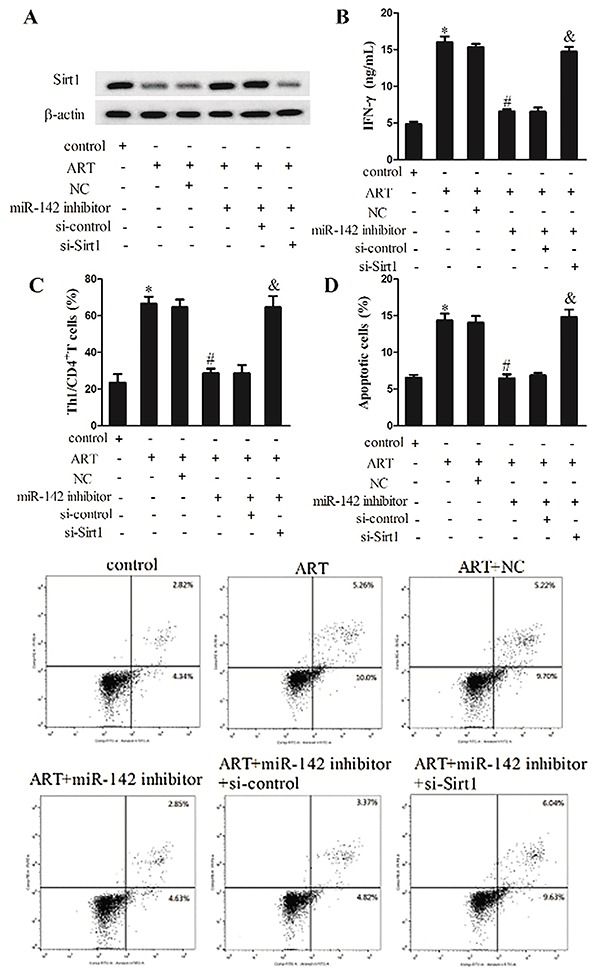
Artesunate (ART, 50 μg/mL) enhanced the pro-apoptotic effects of Th1 cells on ovarian cancer via miR-142/Sirt1 pathway. **A**, Expression of Sirt1 protein was determined using western blot. **B**, Interferon (IFN)-γ level in culture supernatant was evaluated by ELISA. **C**, The percentage of Th1/CD4^+^T cells was analyzed using flow cytometry. **D**, After co-cultured with the differentiation-induced and artesunate-treated CD4+T cells, ID8 cells apoptosis was measured using flow cytometry. NC: negative control. Data are reported as means±SD. *P<0.05 compared to control; ^#^P<0.05 compared to ART+NC; ^&^P<0.05 compared to ART+ miR-142 inhibitor+si-control (ANOVA).

With the positive role of artesunate in Th1 differentiation revealed, we subsequently examined its impact on ovarian cancer cell apoptosis. CD4^+^ T cells were induced for Th1 differentiation, treated with artesunate (ART, 50 μg/mL), and then transfected with miR-142 inhibitor (NC served as control) or si-Sirt1 (si-control served as control). They were divided into 6 groups: control, ART, ART+NC, ART+miR-142 inhibitor, ART+miR-142 inhibitor+si-control, and ART+miR-142 inhibitor+si-Sirt1. The level of Sirt1 protein and IFN-γ, and the Th1/CD4^+^ T cells percentage was determined separately. As displayed in Figure 4A, Sirt1 protein expression was suppressed by artesunate but rescued by miR-142 inhibitor, but it was further reversed by si-Sirt1 transfection. The IFN-γ level was increased by artesunate, which was then reduced with miR-142 inhibitor transfection, but it was largely elevated after Sirt1 silence (Figure 4B). The Th1/CD4^+^ T cells percentage was also increased by artesunate but decreased by miR-142 inhibitor, which was further increased by si-Sirt1 transfection (Figure 4C). After co-culture with the differentiation-induced and artesunate-treated CD4^+^ T cells, apoptosis of ID8 cells was evaluated. The results indicated that artesunate stimulated ID8 cells apoptosis, while miR-142 inhibitor repressed apoptosis but it was then promoted by si-Sirt1 (Figure 4D). These findings illustrated that artesunate enhanced the pro-apoptotic effects of Th1 cells in ovarian cancer through the miR-142/Sirt1 pathway.

## Discussion

Based on the fact that artesunate alters the sensitivity of ovarian cancer cells to chemotherapy and its influence on the function of Th1 cells to secrete IFN-γ, we revealed the role of artesunate in Th1 differentiation and in apoptosis of ovarian cancer cells. It can be summarized that artesunate promoted Th1 differentiation from CD4^+^ T cells by down-regulating Sirt1 through miR-142, thereby enhancing cell apoptosis in ovarian cancer. Our study showed the impact and regulatory mechanism of artesunate on ovarian cancer cell apoptosis, providing novel insights into developing effective therapeutics to improve the outcomes of ovarian cancer.

Artesunate exhibits great anti-tumor properties by regulating different signaling pathways, thus affecting the curative effect of many cancer therapies. In HeLa cells, artesunate combined with irradiation increased cell apoptosis and G2/M cell cycle arrest, and the tumor growth of xenografts from HeLa was also suppressed, suggesting that artesunate increased radiosensitivity of cervical cancer cells (12). By decreasing transforming growth factor β1 (TGF-β1) and IL-10, artesunate reversed the immunosuppression from Colon26 and RKO colorectal cancer cells (23), while it exerted an anti-immunosuppressive effect on cervical cancer by repressing PGE2 production and Foxp3 expression, making it a promising drug for immunotherapy of cervical cancer (13). Now that the artesunate sensitization of ovarian cancer cells to cisplatin has been verified (5), we highlighted that artesunate promoted Th1 cells differentiation, which contributed to cell apoptosis in ovarian cancer. This study demonstrated the inhibitory effects of artesunate on ovarian cancer and reconfirmed the function of Th1 cells and IFN-γ in fighting against cancer, offering an essential reference and perspective for immunotherapy development.

In another aspect, artesunate functioned by interacting with non-coding RNAs to regulate gene expression in cancer cells, such as microRNAs (miRNAs). By up-regulating miR-16 expression, artesunate inhibited COX-2 expression and PGE2 production, which induced apoptosis of bladder cancer cells (21). Similarly, artesunate up-regulated miR-34a expression in a dose-dependent manner, a well-known tumor suppressive miRNA, correlating with reduced CDK4 level in breast cancer cells (22). The current study proved a promotion effect of artesunate on miR-142 expression, which led to restrained expression of Sirt1. MiR-142 functions as a tumor suppressor with diverse targets in many cancers, as it repressed the tumorigenicity of human breast cancer stem cells via activating the WNT signaling pathway (24). Through targeting HSP70, miR-142 inhibited pancreatic cancer cell proliferation, showing its potential target for pancreatic ductal adenocarcinoma therapeutic intervention (25). Even up-regulation of miR-142 in ovarian cancer patients has been observed (19); its role in ovarian cancer initiation and development was clarified in the present study. We elucidated that up-regulation of miR-142 in ovarian cancer promoted Th1 differentiation to induce tumor cell apoptosis via controlling Sirt1, which was closely associated with cell growth, proliferation, differentiation, and apoptosis.

Sirt1 is a highly conserved NAD^+^-dependent deacetylase that is widely expressed in various cells and implicated in growth development, energy metabolism, and cancer progression (26). Previous study has reported that high level of Sirt1 was correlated with ovarian cancer tumorigenesis (27), and over-expression of Sirt1 in epithelial ovarian cancer contributed to chemoresistance and indicated poor prognosis (17). In addition, the roles of Sirt1 in driving Th1 development from differentiation of CD4^+^ T cells and stimulating IFN-γ secretion through in cells have been revealed (16,28). This study initially combined the functions of Sirt1 in regulating ovarian cancer progression and Th1 differentiation, underscoring that down-regulation of Sirt1 contributed to Th1 differentiation and subsequent ovarian cancer cell apoptosis.

In conclusion, the findings of this study illustrated that artesunate promotes Th1 differentiation from CD4^+^ T cells by down-regulating Sirt1 through miR-142, thereby enhancing cell apoptosis in ovarian cancer. This study elucidated the action mechanism of artesunate in the fight against ovarian cancer through influencing Th1 differentiation, providing effective targets for developing novel chemotherapy to improve the outcomes of ovarian cancer.
